# Combination Strategy of Genetic Dereplication and Manipulation of Epigenetic Regulators Reveals a Novel Compound from Plant Endophytic Fungus

**DOI:** 10.3390/ijms23073686

**Published:** 2022-03-28

**Authors:** Lu Yang, Guangwei Wu, Fanyue Meng, Huomiao Ran, Wenbing Yin, Wei Li, Xiaoqing Liu

**Affiliations:** 1 College of Life Science, Capital Normal University, Beijing 100048, China; 2200802093@cnu.edu.cn (L.Y.); 2190802094@cnu.edu.cn (F.M.); 2 Jiangsu Co-Innovation Center of Efficient Processing and Utilization of Forest Resources, Jiangsu Key Lab of Biomass-Based Green Fuels and Chemicals, College of Chemical Engineering, Nanjing Forestry University, Nanjing 210037, China; gweiwu@163.com; 3 State Key Laboratory of Mycology, Institute of Microbiology, Chinese Academy of Sciences, Beijing 100101, China; ranhm@im.ac.cn (H.R.); yinwb@im.ac.cn (W.Y.); 4 Savaid Medical School, University of Chinese Academy of Sciences, Beijing 100049, China

**Keywords:** genetic dereplication, epigenetic regulation, secondary metabolites, oxidative stress, filamentous fungi

## Abstract

The strategies of genetic dereplication and manipulation of epigenetic regulators to activate the cryptic gene clusters are effective to discover natural products with novel structure in filamentous fungi. In this study, a combination of genetic dereplication (deletion of pesthetic acid biosynthetic gene, *PfptaA*) and manipulation of epigenetic regulators (deletion of histone methyltransferase gene *PfcclA* and histone deacetylase gene *PfhdaA*) was developed in plant endophytic fungus *Pestalotiopsis fici*. The deletion of *PfptaA* with *PfcclA* and/or *PfhdaA* led to isolation of 1 novel compound, pestaloficiol X (**1**), as well as another 11 known compounds with obvious yield changes. The proposed biosynthesis pathway of pestaloficiol X was speculated using comparative analysis of homologous biosynthetic gene clusters. Moreover, phenotypic effects on the conidial development and response to oxidative stressors in the mutants were explored. Our results revealed that the new strain with deletion of *PfcclA* or *PfhdaA* in Δ*PfptaA* background host can neutralise the hyperformation of conidia in the *PfptaA* mutant, and that the Δ*PfptaA* Δ*PfhdaA* mutant was generally not sensitive to oxidative stressors as much as the Δ*PfptaA* Δ*cclA* mutant in comparison with the single mutant Δ*PfptaA* or the parental strains. This combinatorial approach can be applied to discover new natural products in filamentous fungi.

## 1. Introduction

Filamentous fungi produce clinically important secondary metabolites (SMs), including many natural products developed into pharmaceutical drugs. The biosynthesis genes for these SMs are usually clustered in a chromosome, and are called biosynthetic gene clusters (BGCs) [1]. Under laboratory cultural conditions, most of BGCs in silence are unexpressed or little expressed. In recent years, many strategies have been developed to effectively activate the silent expressed BGCs for the discovery of new natural products in filamentous fungi, including heterologous expression, promoter engineering, genetic dereplication, modulations of transcription factor, global regulator, epigenetic regulator, and combinational strategies [2,3].

Genetic dereplication is a powerful approach to discover novel compounds from unknown biosynthesis pathways. Eliminating major SMs is particularly effective to increase the odds of detecting minor SMs as well as being useful for the heterologous expression of biosynthetic genes from other fungal species [4]. Deletion of eight of the most highly expressed secondary metabolites gene clusters in *Aspergillus nidulans* resulted in the discovery of aspercryptin [4]. A genetic dereplication approach led to discovery of two novel polycyclic lactones, three new sesquiterpenes, and the known fusidilactone A from *Trichoderma hypoxylon* [5,6]. The biosynthesis gene cluster of atranorin in lichens was identified through genetic dereplication in *Cladonia* and heterologous expression in *Ascochyta rabiei* [7]. A metabolic shunting strategy by deleting the key gene for rubratoxins (with the high yield) biosynthesis combining with the optimization of culture conditions successfully activated multiple silent genes encoding for other polyketide synthases (PKSs), and led to the discovery of 23 new compounds in *Penicillium dangeardii* [8].

Epigenetic regulation also has been proved as an efficient activation approach to access chemical diversity and discover new natural products in filamentous fungi. Deletion or overexpression of histone-modifying enzyme-genes can trigger the expression of the BGCs located in the chromatin [2]. These modifying enzymes catalyze different modifications of histone, including acetylases, methyltransferases, demethylases, deacetylases, phosphorylase, and ubiquitin enzyme, respectively [3]. Modulation of histone acetylation is typically associated with transcriptional activation in different fungal species. Deletion of the histone deacetylase-encoding gene *hdaA* could positively and negatively regulate the production of secondary metabolites in *Aspergillus fumigatus* [9], *Fussarium fujikuroi* [10] and *Pestalotiopsis microspore* [11]. Deletion of an *hdaA* homolog increased production of penicillin and sterigmatocystin in *A. nidulans* [12], melanin in *Magnaporthe orzyae* [13], trichothecenes in *Fusarium asiaticum* [13], pigment in *Aspergillus niger* [14]. Meleagrin/roquefortine alkaloid production was upregulated by 84.8-fold in the *Penicillium chrysogenum* Δ*hdaA* strain [15]. Disruption of *hdaA* resulted in production of four novel natural products as well as deviant growth and physiologic function in *Calcarisporium arbuscular* [16], and production of unknown metabolites in *P. chrysogenum* [17]. In addition, histone methylation through CclA also takes part in the regulation of SMs in fungi. Deletion of *cclA* in *A. nidulans* activated the production of two polyketides F9775A and B as well as emodin analogues [18]. Deletion of a *cclA* homolog resulted in increase of several SMs in *A. fumigatus* [19] and *Aspergillus oryzae* [20], and production of new representatives in *Colletotrichum higginsianum* [21], respectively. Deletion of the *cclA* homolog that encodes for CCL1 led to the increase or loss of various SM production in *Fusarium graminearum* [22] and *F. fujikuroi* [23]. Furthermore, a combinational strategy based on epigenetic regulation provides ways to explore the influence on the secondary metabolism in filamentous fungi [3]. For instance, histone deacetylases HosA and HdaA affect the phenotype and transcriptomic and metabolic profiles in *A. niger*, and especially the yield of fumonisin was obviously reduced in the *A. niger* Δ*hosA* Δ*hdaA* mutant [14]. Deletion of both *cclA* and *sumO* (the ubiquitin-like modifier) led to obvious changes of the colony and a medium color, which reflects an impaired secondary metabolism in *A. nidulans* [24].

The plant endophytic fungi from *Pestalotiopsis* genus is well-studied for their SM production [25,26,27]. *Pestalotiopsis fici* has been reported to produce about 100 distinctive compounds by traditional isolation and genetic manipulation strategies [28,29,30,31,32]. Disruption of epigenetic regulators PfcclA and PfhdaA led to the identification of 15 new structures [29]. The diphenyl ether pestheic acid and its analogues are the major SMs produced in *P. fici* [33]. Disruption of pestheic acid biosynthesis gene (*pta*) could observably reduce the SMs background in this strain [28]. In this study, Δ*Pfpta* Δ*PfcclA* and Δ*Pfpta* Δ*PfhdaA* mutants in *P. fici* were constructed. One novel structure was characterized with 11 known compounds in both of the double deletion mutants, and the effect of these genetic manipulation on the growth, sporulation, and sensitivity to oxidative stress were evaluated.

## 2. Results and Discussion

### 2.1. Construction of PfptaA Deletion Mutants

**Figure 1:**
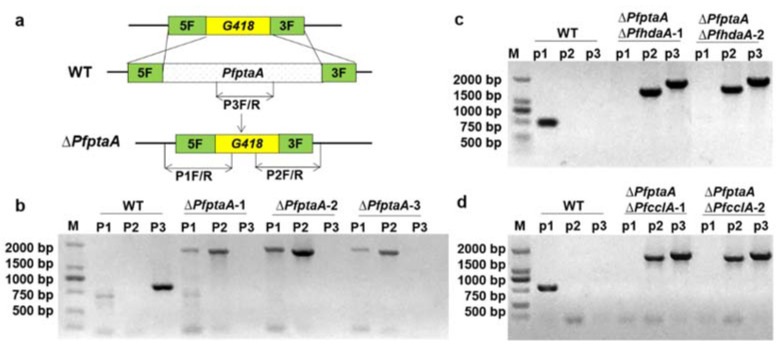
Generation of gene deletion strains. (**a**) Schematic illustration for disruption of *PfptaA* gene in *P. fici*; (**b**) PCR verification for *PfptaA* gene deletion in *P. fici* wild-type strain. The primer pairs of P1F/R, P2F/R, P3F/R, were designed for screening and the products should be 850, 1850, 1780 bp, respectively; (**c**) PCR verification for *PfhdaA* gene deletion in Δ*PfptaA* host; (**d**) PCR verification for *PfcclA* gene deletion in Δ*PfptaA* host.

In previous studies, the *pta* gene cluster for pestheic acid biosynthesis has been identified in *P. fici* [28], and both Δ*PfcclA* and Δ*PfhdaA* mutants has been obtained, respectively [29]. Here, we constructed the single knockout mutant of *PfptaA* (*PFICI_10824*), double knockout mutants of *PfptaA* and *PfcclA* (*PFICI_05127*), and *PfptaA* and *PfhdaA* (*PFICI_08988*) using the previously described transformation method, individually [29] (Figure 1 and Table S1). For deletion of *PfptaA*, we constructed a plasmid containing the upstream and downstream homologous arms of *PfptaA* with the resistance gene of G418 antibiotic. The fragments of deletion cassette of *PfptaA* were amplified by PCR, and the plasmid construct was transformed into the *P. fici* wild type (WT) strain. Then *PfptaA* deletion mutants were verified by diagnostic PCR analysis using designated primers (Figure 1a,b and Table S2). Subsequently, the *PfptaA* deletion plasmid construct was transformed into strains of TYXW7 (Δ*PfcclA*) and TYXW8 (Δ*PfhdaA*). The genomic DNAs of transformants were extracted and correct mutants were verified by diagnostic PCR using designated primers (Figure 1c,d; Figure S1 and Table S2).

### 2.2. Assessment of Secondary Metabolites

**Figure 2:**
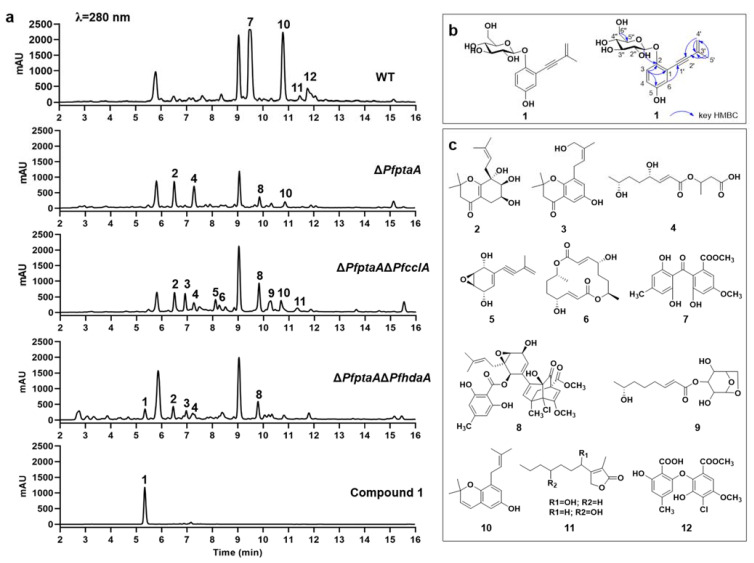
Secondary metabolites analysis for *P*. *fici* wild type and mutants. (**a**) HPLC analysis of secondary metabolites; (**b**) structure and key HMBC correlation of pestaloficiol X (**1**); (**c**) known SMs produced by *P. fici* strains in this study.

To evaluate the modulation of secondary metabolites production via the deletion of the target genes, the strains were grown on a rice-based medium and the culture extracts were analyzed by HPLC and LC-MS. The results were changes of SM production profile in Δ*Pfpta* Δ*PfcclA* and Δ*Pfpta* Δ*PfhdaA* mutants, compared with WT and Δ*Pfpta* strains (Figure 2a). A new peak was obviously detected along with 11 known SMs. Subsequently, the new peak was further isolated and purified to obtain pure compound **1**, produced in Δ*Pfpta* Δ*PfhdaA* mutant (Figure 2b). Compounds **7** and **12** produced in WT were disappeared in both Δ*Pfpta* Δ*PfcclA* and Δ*Pfpta* Δ*PfhdaA* mutants. Compound **10** produced in WT was obviously decreased in both of the double-deletion strains. Compounds **2**, **5**, **8**, and **9** were increased in the deletion strains, in comparison with WT. Compounds **3** and **4** were novel peaks in both of the double deletion strains compared with WT, and compound **4** also was produced in Δ*Pfpta* mutant. The known SMs identified in *P. fici* wild type, Δ*PfcsnE* (the fifth subunit of COP9 signalosome, CsnE), and Δ*PfhdaA* strains are pestaloficiol M (**2**), pestaloficin D (**3**), ficiolide J (**4**), asperpentyn (**5**), ficiolide C (**6**), isosulochrin (**7**), chloropupukeananin (**8**), ficiolide K (**9**), pestaloficiol J (**10**), hydroxyisoseiridin (**11**), and pestheic acid (**12**) [29,33,34,35,36,37,38] (Figure 2c). All of these known structures were elucidated by comprehensive analysis with spectroscopy and HR-ESI-MS (Figures S8–S18).

### 2.3. Identification of Compound **1**

The new compound **1** was only produced in Δ*Pfpta* Δ*PfhdaA* mutant (Figure 2a and Figure S7). To elucidate the structure of **1**, we made a scale-up fermentation and isolated it by a combination of UV-guided fractionation and retention time. Compound **1** was isolated as yellowish oil with the molecular formula C_17_H_20_O_7_, which was deduced by the protonated molecule HR-ESI-MS ion peak with [M + Na]^+^ at *m*/*z* 359 (Figure S7). The careful inspection of ^1^H and ^13^C NMR of **1** with previously identified compounds in fungal strains indicated that **1** was a 1, 3-enynes-based cyclohexanoid terpenoids. Further analysis of 1D NMR data provided the evidence that backbone of **1** is identical to siccayne which was first isolated as an antibiotic compound from the deuteromycete *Helminthosporium siccans* in 1981 [39]. The remaining NMR signals and unsaturation revealed that the hexose moiety is proposed. H-1′′/C-5′′ and H-5′′/C-1′′ HMBC correlations established that the hexose moiety underwent ring closure. The key HMBC correlation of H-1′′ with C-2 allowed us to assemble the intact planar structure by ether bond between the siccayne and hexose moiety (Figure 2b; Table 1 and Figures S2–S6). Unfortunately, due to the lower content of **1**, the configuration of hexose moiety remained unclear. Finally, compound **1** was determined to be a new structure and named pestaloficiol X. Glycosylation of small molecular plays a significant role in drug discovery and development [40]. As the precursor of **1**, siccayne exhibited moderate antibiotic activity of Gram-positive bacteria and some fungal strains [39], and cytotoxic activity against multiple human cancer cell lines [41]. The glycosylated siccayne such as **1** was speculated to improve the solubility in water and druggability.

### 2.4. Proposed Biosynthesis Pathway of **1** in P. fici

**Figure 3:**
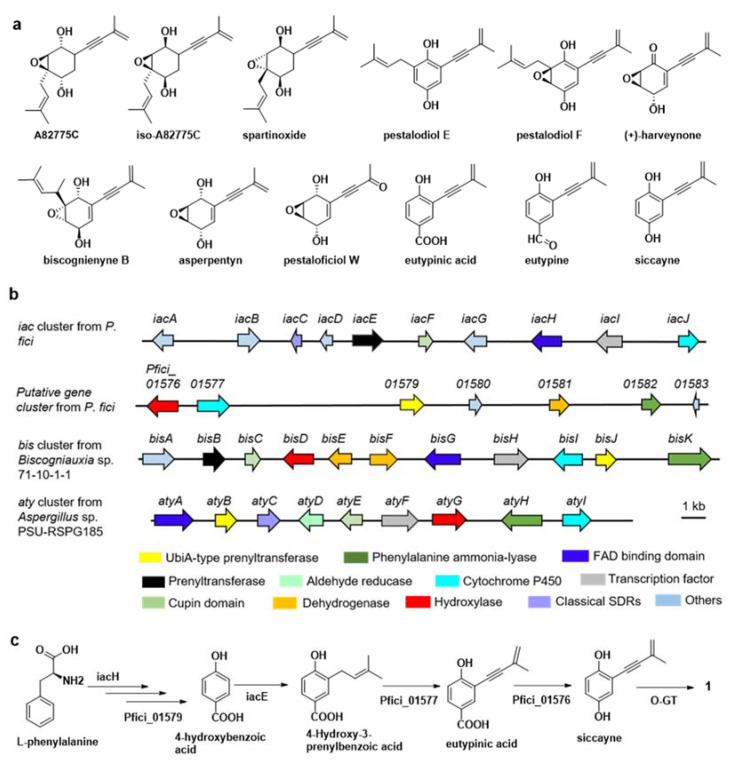
The structure analogues with alkynyl of **1** and biosynthesis of **1**. (**a**) The structure analogues of **1** produced in filamentous fungi; (**b**) the gene clusters related to biosynthesis of siccayne and **1** in filamentous fungi; (**c**) proposed biosynthetic pathway for **1** in *P. fici*.

Alkyne is a typical active group in many natural products with antitumor and anti-HIV activities. There have been several analogues of **1** with alkynyl pattern isolated in *P. fici* [29,33], *Biscogniauxia* sp. [42], *Eutypa lata* [43] and *Aspergillus* sp. [44] (Figure 3a). The biosynthesis gene cluster *iac* for iso-A82775C has been identified in *P. fici*. While the prenyltransferase IacE is responsible for the modification of isopentenyl, the mechanism of alkyne formation in the biosynthetic pathway was not elucidated in the work [45]. Inspired by this research, the *bis* gene cluster for biscognienyne B biosynthesis was identified in *Biscogniauxia* sp., in which cytochrome P450 enzyme (BisI) is confirmed to catalyze the alkynylation of the prenyl chain [46]. Surprisingly, there was no homologous gene of *bisI* in *iac* gene cluster in *P. fici*. In addition, the oxidoreductase gene *iacJ* in the *iac* cluster was unrelated to the formation of alkyne [45]. Simultaneously, another P450 monoxygenase (AtyI) was verified to catalyze dehydrogenation of the prenyl chain and to yield an alkene moiety in compound asperpentyn in *Aspergillus* sp. [47]. The cytochrome P450 gene *Pfici_01577* located outside of *iac* gene cluster in *P. fici* was found by BlastP analysis to be the cytochrome P450 homologous protein of both BisI and AtyI with identity and similarity of both 89%/79% and 78%/87%, respectively (Figure 3b) [46,47]. So, there is an obvious difference in the biosynthetic gene clusters in different fungal species that produced siccayne and its analogues. In addition, glycosylation of natural products is catalyzed by glycosyltransferases (GTs). A phenolic GT MhGT1 identified in *Mucor hiemalis* exhibited broad substrate scope and regio- and stereospecificy [48]. The several O-GTs predicted in *P. fici* were not within the gene clusters and distributed throughout the genome [40]. The biosynthesis of **1** was proposed according to the above-mentioned studies. Compound **1** was synthesized via the biosynthesis pathway of *iac* gene cluster along with a putative cytochrome P450 enzyme (Pfici_01577), a putative hydroxylase (Pfici_01576), and an unknown glycosyltransferase in *P. fici*. The proposed biosynthetic pathways of pestaloficiol X (**1**) in *P. fici* was shown in Figure 3c.

### 2.5. Assessment of Conidia Development in the Mutant Strains

**Figure 4:**
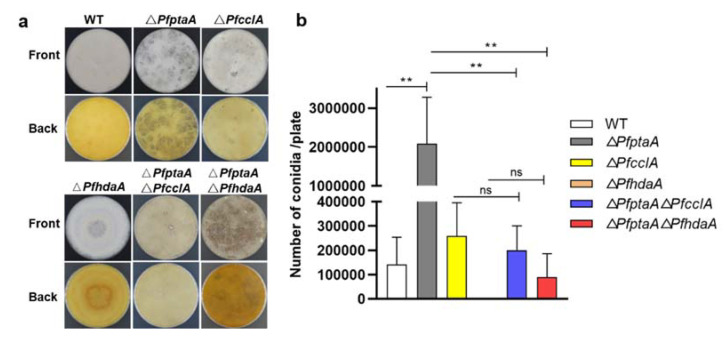
Phenotypic effect on conidia development in *P. fici* strains. (**a**) Phenotype observations of strains of *P. fici* WT, Δ*PfptaA*, Δ*PfcclA*, Δ*PfhdaA*, Δ*PfptaA* Δ*PfcclA*, and Δ*PfptaA* Δ*PfhdaA*; (**b**) the difference of conidia production in strains of *P. fici* WT, Δ*PfptaA*, Δ*PfcclA*, Δ*PfhdaA*, Δ*PfptaA* Δ*PfcclA*, and Δ*PfptaA* Δ*PfhdaA*. All of the strains were grown on PDA plates for number determination at 25 °C for 14 days. Three replicates were done for each culture of strain. Error bars represent the standard deviations. Asterisks indicated significant differences in mean values (*p* < 0.01(**); *p* > 0.05 (ns)).

Deletion of the *cclA* can observably decrease the amount of asexual spores and block the production of mature fruiting bodies and sexual development in *A*. *nidulans* [24]. To examine the effect on the morphology and conidia development, the strains of *P. fici* WT, Δ*PfptaA*, Δ*PfcclA*, Δ*PfhdaA*, Δ*PfptaA* Δ*PfcclA*, and Δ*PfptaA* Δ*PfhdaA* were cultivated on Potato Dextrose Agar (PDA) plate. The differences in morphology between the mutants and WT strain were shown, respectively (Figure 4a), and the differences in conidia number among all of the strains were analyzed (Figure 4b). In the Δ*PfptaA* mutant, the conidia number increased about 10-fold compared with *P. fici* WT. Both deletion of *PfcclA* and *PfhdaA* led to no obvious change in conidia number compared with *P. fici* WT. This suggested that *PfptaA* is involved in the conidia development and formation, and *PfcclA* and *PfhdaA* has little effect on conidia development and formation. Furthermore, deletion of *PfcclA* and *PfhdaA* in Δ*PfptaA* mutant had no differential effect on the conidia number compared with Δ*PfcclA* and Δ*PfhdaA* mutants, respectively, but both double mutant strains had decreased numbers of conidia in comparison with Δ*PfptaA* mutant. The results indicated that deletion of *PfcclA* or *PfhdaA* neutralises the enhancement of conidia formation in Δ*PfptaA* host, suggesting interconnected regulatory network among these genes and products in conidia development and formation.

### 2.6. Assessment of Oxidative Stress Response of the Mutant Strains

**Figure 5:**
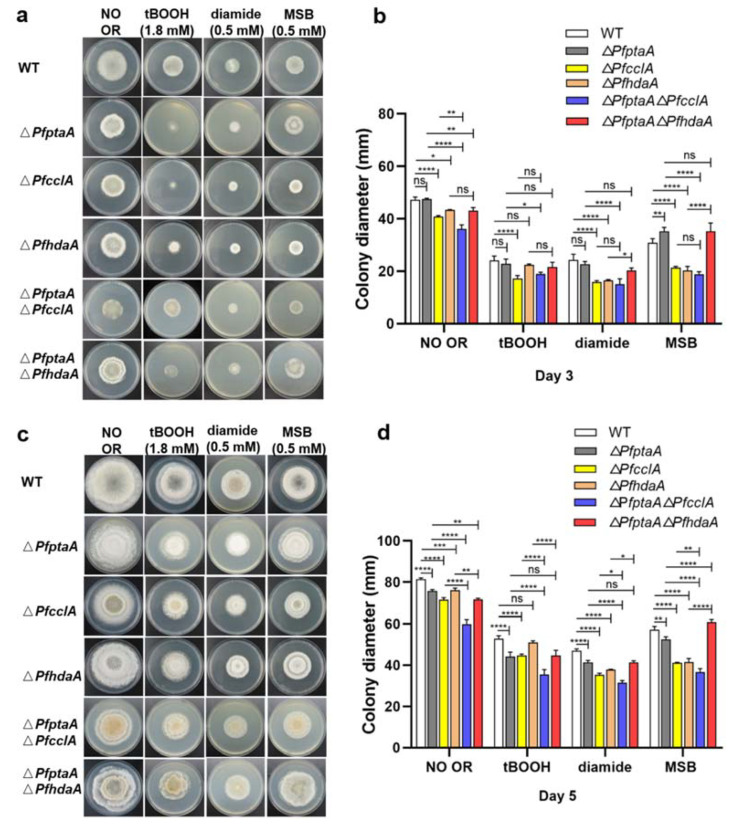
Comparison of oxidative stress tolerances of *P. fici* strains. (**a**) Mycelia growth of the mutants under oxidative stress on the 3rd day; (**b**) the colony diameters of the testing strains were measured on the 3rd day; (**c**) mycelia growth of the mutants under oxidative stress on the 5th day; (**d**) the colony diameters of the testing strains were measured on the 5th day. The spores of WT strain and mutants were inoculated on PDA media with or without tBOOH (1.8 mM), diamide (0.5 mM), or MSB (0.5 mM), and cultured at 25 ℃ for 5 days. Three replicates were done for each culture of strain. OR is the abbreviation of oxidative reagents. Error bars represented the standard deviations. Asterisks indicate significant differences in mean values (*p* < 0.0001 (****), *p* < 0.001 (***), *p* < 0.01 (**), *p* < 0.05 (*)).

Epigenetic regulators also influence the fungal growth, development, infection, and their adaptation to environment. For instance, the loss of *hdaA* did not affect the growth rate of *A. nidulans* [49], but *A. fumigatus* Δ*hdaA* strain showed a statistically significant reduction of growth compared with the wild type [9]. HdaA was involved in sclerotia formation in *A*. *flavus* [50], and the deletion of *hdaA* reduced the oxidative stress tolerance of *A. nidulans* [49]. On the other hand, the deletion of *cclA* strongly reduced mycelial growth, asexual sporulation and spore germination, but did not impair the morphogenesis of specialized infection structures in *C. higginsianum* [21]. To assess any impact of target genes on oxidative stress response, *P. fici* WT strain and all of mutants were subjected to three oxidative reagents, including diamide, tert-butylhydroperoxide (tBOOH), and menadionesodium bisulfite (MSB). Separate deletion of *PfptaA*, *PfcclA*, and *PfhdaA*, partly lowered the growth rate on PDA medium, and the inhibition was more obvious in the Δ*PfcclA* mutant than the Δ*PfptaA* and Δ*PfhdaA* mutants. There were obvious differences of sensitivity to tBOOH, diamide, and MSB in different mutants in the third day and fifth day as determined via the measurement of colony diameter. The smaller colony size might indicate the more sensitivity. Almost all of the mutants were more sensitive to tBOOH, diamide, and MSB than *P. fici* WT, and displayed slower growth in the presence of oxidative stressors besides the Δ*PfptaA* Δ*PfhdaA* mutants treated with MSB (Figure 5a–d). Moreover, the MSB effects on colony size on the 3rd day and the 5th day were opposite between the *P. fici* WT and Δ*PfptaA* mutant. For the double genes-deficient mutants, the Δ*PfptaA* Δ*PfcclA* mutant was more sensitive to all of the stressors than the Δ*PfptaA* mutant, and only more sensitive to tBOOH than Δ*PfptaA*. The Δ*PfptaA* Δ*PfhdaA* mutant was not sensitive to MSB as well as Δ*PfptaA* mutant, and it was apparently not as sensitive as the Δ*PfhdaA* mutant to MSB. The effects on strain sensitivity to the oxidative stress agents were generaly different by deletion of epigenetic regulators in various fungal species. The *A. nidulans* Δ*hdaA* mutant increased susceptibility to oxidative stress compared with the wild type [49], but the growth of the *A. fumigatus* Δ*hdaA* mutant was not affected under oxidative stress conditions compared with the wild type [9]. Moreover, conidial production was indistinguishable between *A. fumigatus* Δ*hdaA* and wild-type strains [9]. The *A. fumigatus* Δ*cclA* mutant was more sensitive to chemical 6-azauracil (6AU) compared with the wild type [19]. The cause leading to these different effects may be involved in the changes of SMs, and the regulatory mechanism should be explored in the future.

## 3. Materials and Methods

### 3.1. Strains, Media and Culture Conditions

*P. fici* CGMCC3.15140 and its correct transformants were grown at 25 °C on Potato Dextrose Agar (PDA) medium with appropriate antibiotics as required (Table S1). All of the strains were grown on the rice medium with water at 25 °C for 20 days for extraction and analysis of secondary metabolites. *Escherichia coli* DH5α and *Agrobacterium tumefaciens* AGL-1 were propagated at 37 °C in LB medium with appropriate antibiotics for plasmid DNA amplification and transformation, respectively.

### 3.2. Plasmids for Deletion of PfptaA

The plasmids and primers are listed in Table S1 and Table S2, respectively. PCR amplifications were executed in the T100TM Thermal cycler (Bio-Rad, Hercules, CA, USA). TransStart-FastPfu DNA polymerase as a High-Fidelity DNA polymerase (TransGene Biotech, Beijing, China) was used to amplify the gene fragments. PCR screenings for transformants were performed by using 2×Taq Mix kit (Tiangen Biotech, Beijing, China). PCR reaction and thermal profiles were referred to the manufacturer´s instructions. The restriction enzymes used in this work were obtained in New England Biolabs (New England Biolabs Inc. (NEB), Ipswich, MA, USA). To generate the deletion cassette, we used Fusion PCR strategy as described previously [51]. Briefly, G418 was amplified from the pAG1-H3-G418, and around 1.1 kb of fragments upstream and downstream of the gene *PfptaA* were amplified from *P. fici* genomic DNA using the designed primers. The three PCR fragments were ligated into the T-vector p-Blunt, and then were amplified for transformation in *P. fici* strains.

### 3.3. Transformation in P. fici

For creation of deletion of *PfptaA (PFICI_10824*) mutants in *P. fici* wild type, Δ*PfcclA,* and Δ*PfhdaA* mutants, the deletion cassette was amplified with the template of pYYJ1.1 using primers 10824-5f-FL and 10824-3f-RL. The DNA cassette fragments were transformed into *P. fici* WT, TYXW7.1 and TYXW8.1 as described previously [29]. Then candidate colonies were singled out after culturing on PDA with G418 resistant at 28 °C for 5 days. The disruption mutants were verified using diagnostic PCR with primers inside and outside of the gene *PfptaA* (Figure 1a and Table S2).

### 3.4. Oxidative Stress Sensitivity Assays

Different oxidative reagents were used to estimate the stress sensitivity of the mutants according to the method described previously [35]. Moreover, the colony diameters are the indicator supplementing with the following stress-generating agents: 0.5 mM diamide, 0.5 mM menadionesodium bisulfite (MSB), and 1.8 mM tert-butylhydroperoxide (tBOOH), respectively. The strains were incubated at 25 °C for 5 days, and colony diameters were measured on the 3rd day and 5th day. Three replicates were performed for each experiment.

### 3.5. Conidia Counting

The conidia counting in *P. fici* wild type and its mutants were performed as described previously [52]. These strains were grown at 25 °C on PDA medium in 90 mm plate for 14 days. Three replicates were performed for each culture sample. Then, we used ddH_2_O with 0.1% (*v*/*v*) Tween to flush the plates repeatedly and remove the hyphae and impurities through Miracloth. The filtrate containing conidia was centrifuged and concentrated to 1 mL of volume. The number of conidia from different mutants and wild type was determined using a blood-cell-counting plate. Values are means of three replicates for each culture are presented. Data were analyzed using the GraphPad Prism 8.0 performing Tukey–Kramer multiple comparison test at *p* ≤ 0.05. Asterisks indicate statically significant differences in mean values.

### 3.6. Analytical Methods for HPLC and LC-MS

Analysis of secondary metabolites was performed on a Waters HPLC system (Waters 2998, Photodiode Array Detector) with an ODS column (C18, 250_4.6 mm, Waters Pak, 5 μm). Water (A) and acetonitrile (B), both with 0.1 % (*v*/*v*) formic acid, were used as solvents at a flow rate of 1 mL/min. The substances were eluted with a linear gradient from 5–100% B in 40 min, then washed with 100 % (*v*/*v*) solvent B for 5 min and equilibrated with 5 % (*v*/*v*) solvent B for 5 min. UV absorptions at 236 nm were illustrated. LC-MS analyses of secondary metabolites was determined using an Agilent 1200 Accurate-Mass QTOF LC/MS system (Agilent Technologies, Santa Clara, CA, USA) with Agilent ZORBAX Eclipse column (C18 Plus, 2.1 × 4.6 mm, 3 μm) and an electrospray ionization (ESI) source. Water (A) and acetonitrile (B), both with 0.1 % (*v*/*v*) formic acid, were used as solvents at a flow rate of 1 mL/min. The substances were eluted with a linear gradient from 5–100% B in 40 min, then washed with 100 % (*v*/*v*) solvent B for 5 min and equilibrated with 5 % (*v*/*v*) solvent B for 5 min.

### 3.7. Isolation and Identification of New Compound

The Δ*PfptaA* Δ*PfhdaA* mutant was cultivated in flasks. Each flask contained 80 g rice and 120 mL distilled water and was sterilized by autoclave. A total of 10 kilograms of rice media were made, and static cultured at 25 °C for 20 d. The rice culture was extracted three times with ethyl acetate. The organic phase was evaporated to dryness under reduced pressure to afford the residue (7.6 g). The crude residue was applied on a C-18 ODS column using a stepped gradient elution of MeOH-H_2_O yielding 10 subfractions (fractions 1–10). Fraction 2 (eluting with MeOH:H_2_O = 25:75) was chromatographed on Sephadex LH-20 (MeOH) and the targeted fraction 2.1 was further separated by HPLC (C-18ODS) using a stepped gradient elution of MeOH-H_2_O (5:95 to 100:0, 120 min) to furnish eight subfractions 2.1.1–2.1.8. The subfraction 2.1.1 was further purified by semi-preparative HPLC with a gradient of H_2_O and CH_3_CN (linear gradient of 15 % to 45 % CH_3_CN over 30 min at 3 mL/min) to afford compound **1** (1.7 mg tR = 5.4 min). The assignments of 11 compounds were based on the published data of proton LC-MS. The related figures were shown individually, in the same order as they are referred to in the manuscript.

## 4. Conclusions

In summary, we developed a combined approach to modulate the secondary metabolic profile in filamentous fungi. We deleted the epigenetic regulators *PfcclA* or *PfhdaA* in a host lacking major SM pestheic acid. This led to significant changes in secondary metabolic profiles. We discovered 1 novel SM named pestaloficiol X (**1**) as well as 11 other known compounds with obvious yield changes in Δ*PfptaA* Δ*PfcclA* or/and Δ*PfptaA* Δ*PfhdaA* mutants. Compound **1** was only produced in the Δ*Pfpta* Δ*PfhdaA* mutant strain, suggesting a successful activation by modulation of histone acetylation and *Pfpta* dereplication. Moreover, the deletion of *PfptaA* in Δ*PfcclA* or Δ*PfhdaA* background host did not overproduce conidia as seen in the single mutant Δ*PfptaA*. We also found that the Δ*PfptaA* Δ*PfhdaA* mutant is generally not sensitive to oxidative stressors in comparison with the single mutant Δ*PfptaA* or the parental strains, whereas the Δ*PfptaA* Δ*PfcclA* mutant was more sensitive. Our findings support that combination strategy of genetic dereplication and manipulation of epigenetic regulators is an efficient approach to discover novel SMs in plant endophytic fungi *P. fici* as well as is a valuable strategy to be applied for new natural product discovery in filamentous fungi.

## Figures and Tables

**Table 1:** ^1^H (500 MHz) and ^13^C (125 MHz) NMR spectroscopic data for **1** in DMSO-*d*_6_.

Position	*δ*_C_, Type	*δ*_H_, Multi., *J* in Hz	HMBC Correlation	^1^H-^1^H COSY
1	113.0 C	-	-	-
2	150.5 C	-	-	-
3	117.0 CH	6.99, d, 9.8	C-1′, 1, 2, 5	H-4
4	116.8 CH	6.71, m, overlap	C-2, 6	H-3
5	151.7 C	-	-	-
6	118.2 CH	6.72, m, overlap	C-1′, 4, 5	-
1′	85.3 C	-	-	-
2′	94.1 C	-	-	-
3′	126.7 C	-	-	-
4′	122.1 CH_2_	5.38, s 5.35, s	C-2′, 3′, 5′	H-5′
5′	23.2 CH_3_	1.93, s	C-2′, 3′, 4′	H-4′
		-	-	
1″	100.7 CH	4.81, d, 7.2	C-2	H-2″
2″	73.9 CH	3.26–3.22, overlap	C-1″, 4″	H-1″
3″	76.9 CH	3.26–3.12, overlap	C-4″	-
4″	70.1 CH	3.26–3.22, overlap	C-5″	-
5″	77.0 CH	3.26–3.12, overlap	C-4″	-
6″	60.7 CH_2_	3.65, d, 12.0 3.51–3.16, overlap	C-5″	-
-OH		9.39, brd	-	-
-OH	-	4.53, brs	-	-
-OH	-	5.07, brs	-	-
-OH	-	7.40, brs	-	-
-OH	-	8.42, brs	-	-

## Data Availability

All data are contained within this manuscript.

## Supplementary Materials

The following supporting information can be downloaded at: https://www.mdpi.com/article/10.3390/ijms23073686/s1.
