# Supplementary material for: Combination Strategy of Genetic Dereplication and Manipulation of Epigenetic Regulators Reveals a Novel Compound from Plant Endophytic Fungus
Source: Int J Mol Sci. 2022 Mar 28;23(7):3686. doi: 10.3390/ijms23073686 (PMC8998291; doi:10.3390/ijms23073686)
Supplement: Supplementary file 1 [file ijms-23-03686-s001.zip › ijms-1611396-supplementary.pdf]

Supplementary Material

Combination Strategy of Genetic Dereplication and Manipulation of Epigenetic Regulators Reveals A Novel Compound from Plant Endophytic Fungus

Lu Yang[1, 3], Guangwei Wu[2], Fanyue Meng[1], Huomiao Ran[3], Wen-Bing Yin[3, 4], Wei Li[3, 4 *] and Xiaoqing Liu[1 *]

[1] College of Life Science, Capital Normal University, Beijing 100048, P. R. China

[2] Jiangsu Co-Innovation Center of Efficient Processing and Utilization of Forest Resources, College of Chemical Engineering, and Jiangsu Key Lab of Biomass-Based Green Fuels and Chemicals, Nanjing Forestry University, Nanjing 210037, P. R. China

[3] State Key Laboratory of Mycology, Institute of Microbiology, Chinese Academy of Sciences, Beijing 100101, P. R. China

[4] Savaid Medical School, University of Chinese Academy of Sciences, Beijing, 100049, P. R. China

*** Correspondence:**

Corresponding Authors

Email: Wei Li, liw@im.ac.cn; Xiaoqing Liu, liuxq@cnu.edu.cn.

CONTENTS

Table S1. Strains and plasmids used in this study.

Table S2. Primers used in this study.

Figure S1. Schematic illustration of disruption of *PfcclA* and *PfhdaA* in *P. fici* WT strain.

Figure S2. ^{1}H NMR spectrum of **1** in DMSO-d_6 (500 MHz).

Figure S3. ^{13}C NMR spectrum of **1** in DMSO-d_6 (125 MHz).

Figure S4. HSQC spectrum of **1** in DMSO-d_6.

Figure S5. HMBC spectrum of **1** in DMSO-d_6.

Figure S6. ^{1}H-^{1}H COSY spectrum of **1** in DMSO-d_6.

Figure S7. LC-MS analysis of compound **1** in the WT, Δ*PfptaA*, Δ*PfptaA*Δ*PfcclA*, and Δ*PfptaA*Δ*PfhdaA* strains.

Figure S8. LC-MS analysis of compound **2** in the WT, Δ*PfptaA*, Δ*PfptaA*Δ*PfcclA*, and Δ*PfptaA*Δ*PfhdaA* strains.

Figure S9. LC-MS analysis of compound **3** in the WT, Δ*PfptaA*, Δ*PfptaA*Δ*PfcclA*, and Δ*PfptaA*Δ*PfhdaA* strains.

Figure S10. LC-MS analysis of compound **4** in the WT, Δ*PfptaA*, Δ*PfptaA*Δ*PfcclA*, and Δ*PfptaA*Δ*PfhdaA* strains.

Figure S11. LC-MS analysis of compound **5** in the WT, Δ*PfptaA*, Δ*PfptaA*Δ*PfcclA*, and Δ*PfptaA*Δ*PfhdaA* strains.

Figure S12. LC-MS analysis of compound **6** in the WT, Δ*PfptaA*, Δ*PfptaA*Δ*PfcclA*, and Δ*PfptaA*Δ*PfhdaA* strains.

Figure S13. LC-MS analysis of compound **7** in the WT, Δ*PfptaA*, Δ*PfptaA*Δ*PfcclA*, and Δ*PfptaA*Δ*PfhdaA* strains.

Figure S14. LC-MS analysis of compound **8** in the WT, Δ*PfptaA*, Δ*PfptaA*Δ*PfcclA*, and Δ*PfptaA*Δ*PfhdaA* strains.

Figure S15. LC-MS analysis of compound **9** in the WT, Δ*PfptaA*, Δ*PfptaA*Δ*PfcclA*, and Δ*PfptaA*Δ*PfhdaA* strains.

Figure S16. LC-MS analysis of compound **10** in the WT, Δ*PfptaA*, Δ*PfptaA*Δ*PfcclA*, and Δ*PfptaA*Δ*PfhdaA* strains.

Figure S17. LC-MS analysis of compound **11** in the WT, Δ*PfptaA*, Δ*PfptaA*Δ*PfcclA*, and Δ*PfptaA*Δ*PfhdaA* strains.

Figure S18. LC-MS analysis of compound **12** in the WT, Δ*PfptaA*, Δ*PfptaA*Δ*PfcclA*, and Δ*PfptaA*Δ*PfhdaA* strains.

REFERENCES

Table S1. Strains and plasmids used in this study.

**Plasmids/Strains**	**Descriptions**	**References**
Pestalotiopsis fici		
CGMCC3.15140	WT	[1]
AGL-1	*Agrobacterium tumefaciens* AGL-1	[2]
TYXW7.1	Δ*PFICI_05127::hph*	[2]
TYXW8.1	Δ*PFICI_08988::hph*	[2]
TYYJ6	Δ*PFICI_10824*::*G418*	This study
TYYJ10	Δ*PFICI_10824*::*G418;* Δ*PFICI_05127*::*hph*	This study
TYYJ11	Δ*PFICI_10824*::*G418;* Δ*PFICI_08988*::*hph*	This study
pAG1-H3-G418	*G418 in pAG1-H3*	[3]
pYYJ1.1	*PFICI-10824* (5f+G418+3f) in pBlunt	This study

TXX = original transformant, pXX = plasmid

Table S2. Primers used in this study.

Primer name	Oligonucleotide sequence (5'-3')	Use
10824-5f-FL	CAGCTATGACCATGATTACGCCAAGCTTGGAACGCGAAGGAC ATTCGGAC	PFICI_10824 5 flanks amplification
10824-5f-RL	CGTTACTAGTGGATCCGAGCTCGGTACCATACCCTATCAGGTT CGCG	
10824-3f-FL	GAAGTTCCTATTCTCTAGAGTCGACAAGGGCCAGGTACCGATC ATACGTC	PFICI_10824 3 flanks amplification
10824-3f-RL	CCAGTGTGATGGATATCTGCAGAATTGCGAAGCAAGTGGATG GCAGCTG	
G418-F	GTACCGAGCTCGGATCCACTAG	G418 amplification
G418-R	CCTTGTCGACTCTAGAGAATAGGAAC	
10824-5f-EF	CAGGCGGATTGAAGAGTTGG	For verification and screening of PFICI_10824 deletion mutant
10824-TR	GAGCATCATATTCCTGCCGC	
10824-TF	GCATAAACACGCACTATGCC	
10824-3f-ER	CCGATTGCTACCGTAACGAG	
10824 RT-F	GCAGTGCGAGAGGAGATACG	
10824-RT-R	CAAGATCTCACCGACAACTTGC	
05127RT_F	GAACCCAAACCCAGAACC	Verification of PFICI_05127 deletion mutant
05127RT_R	CGCATCCTCGAAGCTCATG	
05127_5f_EF	CGCACAAGTTATCGTGCAC	
05127T_R	CTATGTCAGGCACCCAGTC	
05127T_F	CTCGCCGATAGTGGAAACC	
05127_3f_ER	GACGACCTCAACCTGTC	
08988RT_F	CCCTGGAAAGCCAGACAATATG	Verification of PFICI_08988 deletion mutant
08988RT_R	CTGGTAGTTGCGGATGACGTC	
08988_5f_EF	GAAGGTCCGTTGGTGATG	
08988T_R	GTCCTCGTTCCTGTCTGCTA	
08988T_F	GTCCAAGCAGCAAAGAGTG	
08988_3f_ER	GAGCGGCAATAGAATGAGG	

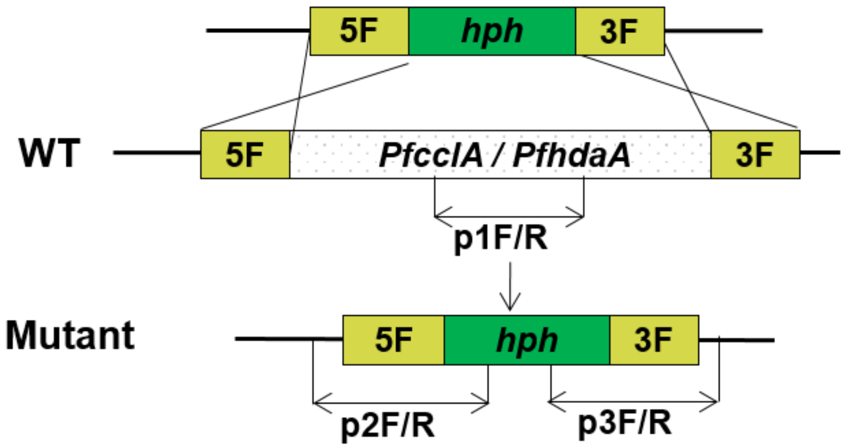

Figure S1. Schematic illustration of disruption of *PfcclA* and *PfhdaA* in *P. fici* WT strain. The primer pairs of P1_F/R, P2_F/R, P3_F/R were designed for verification [2].

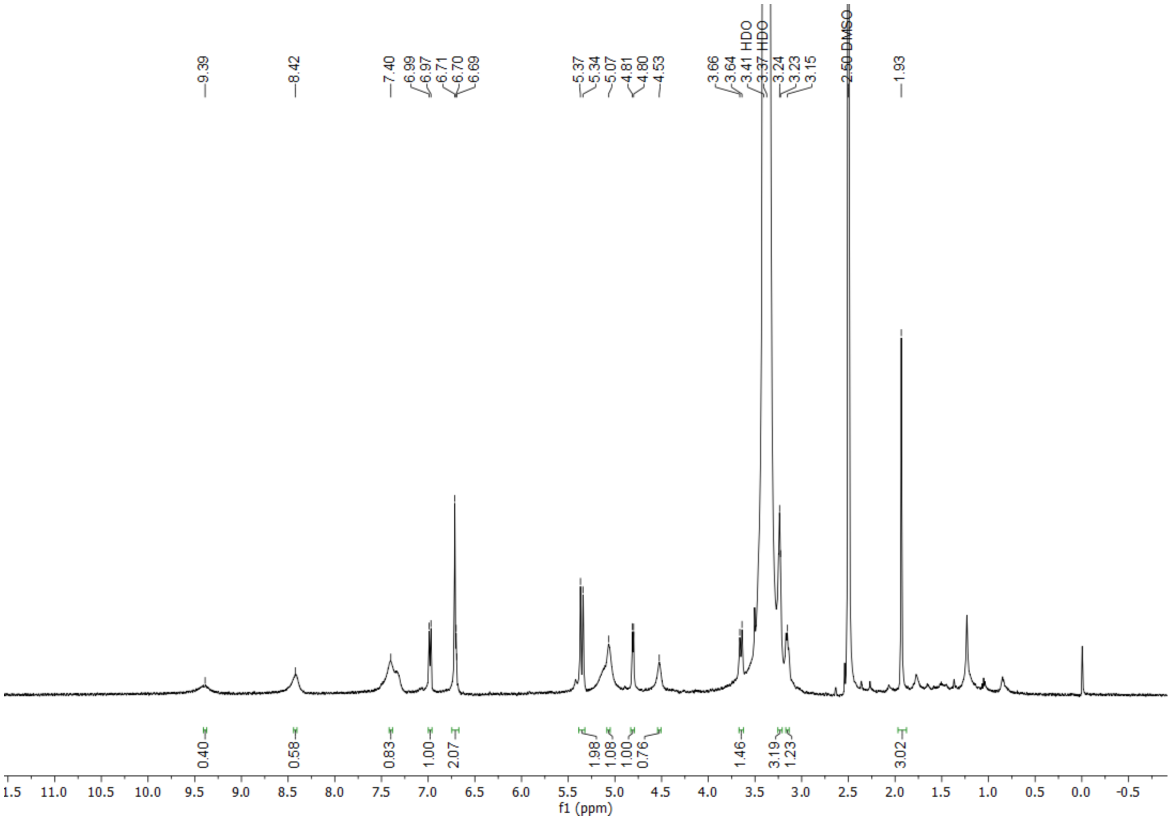

Figure S2. ^{1}H NMR spectrum of **1** in DMSO-d_6 (500 MHz).

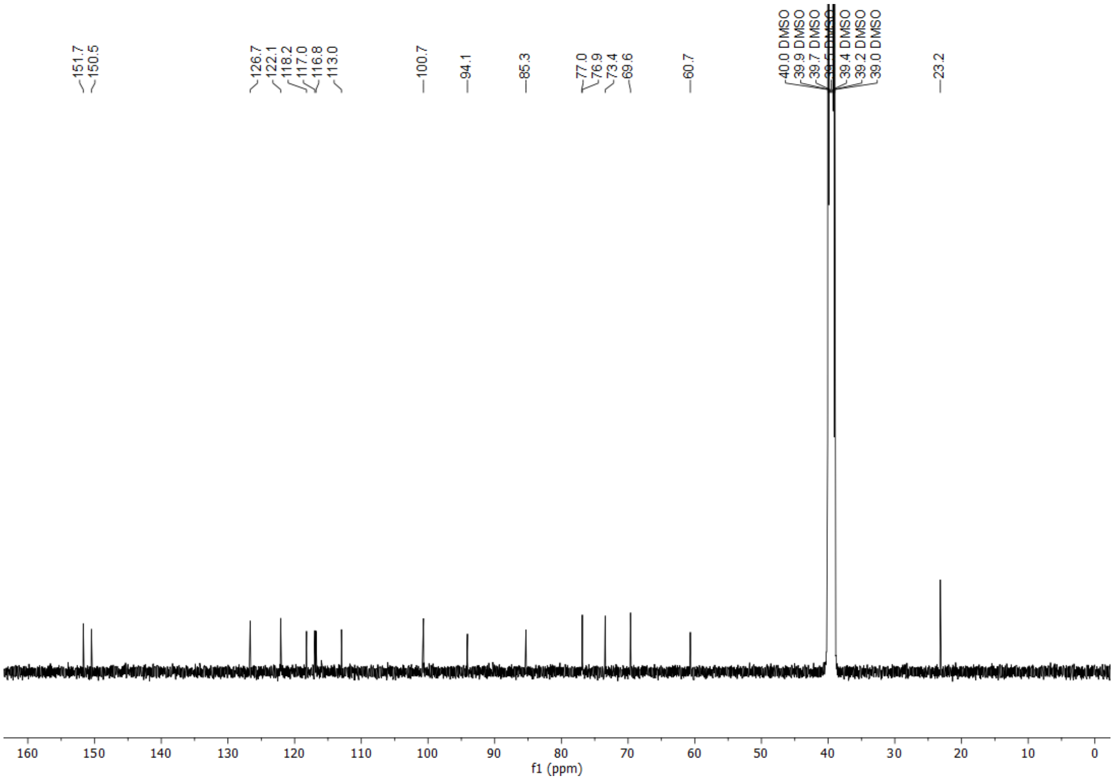

Figure S3. ^{13}C NMR spectrum of **1** in DMSO-d_6 (125 MHz).

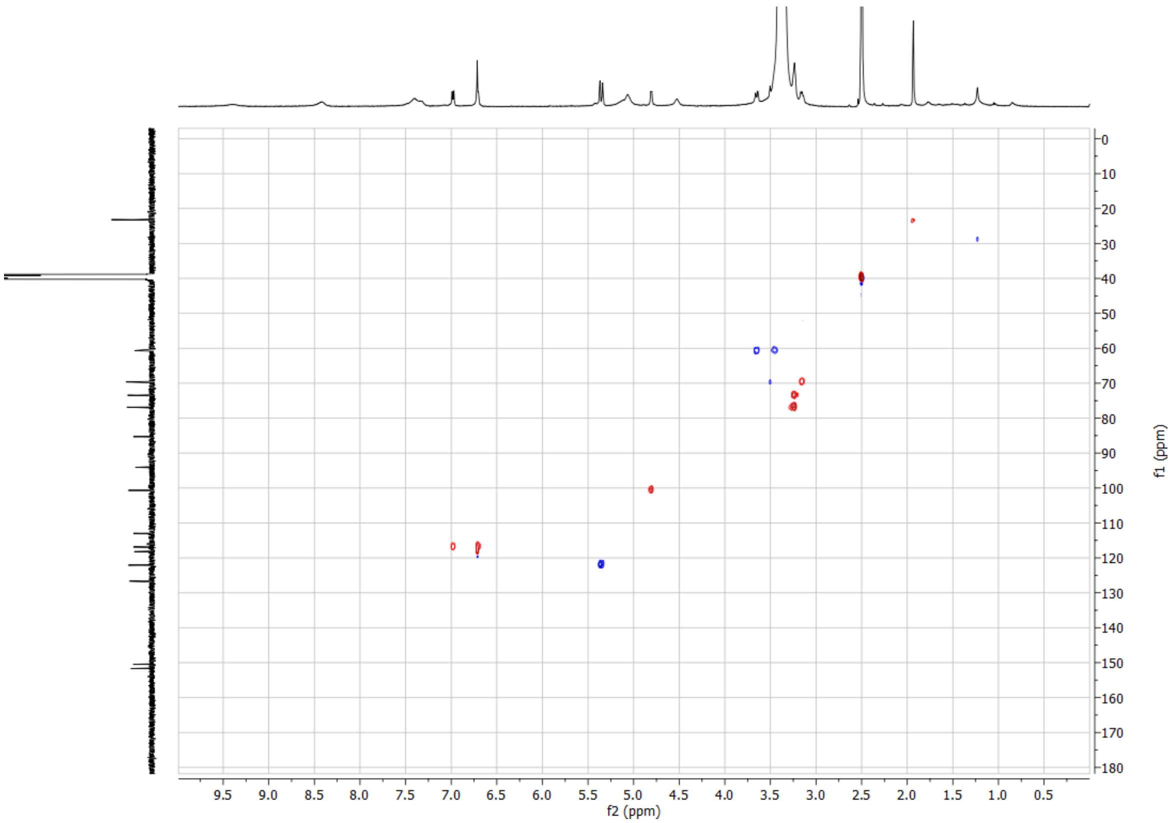

Figure S4. HSQC spectrum of **1** in DMSO-d_6.

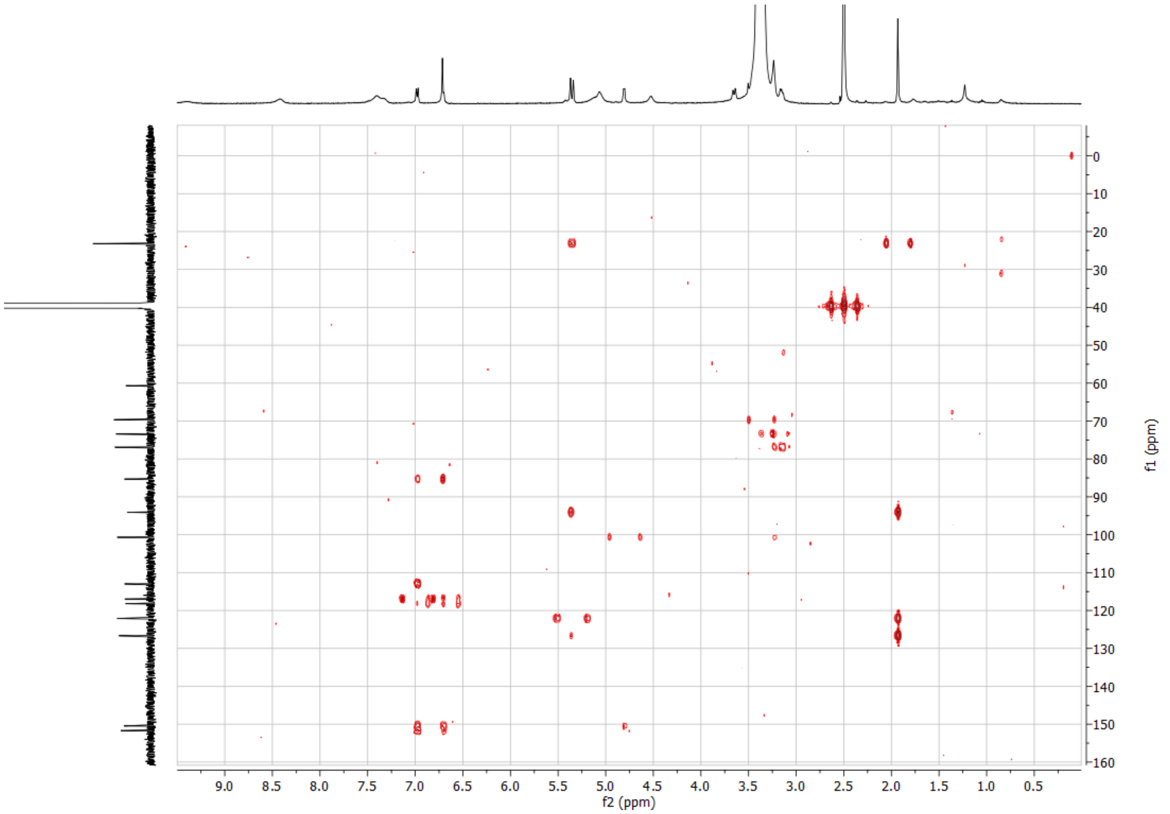

Figure S5. HMBC spectrum of **1** in DMSO-d_6.

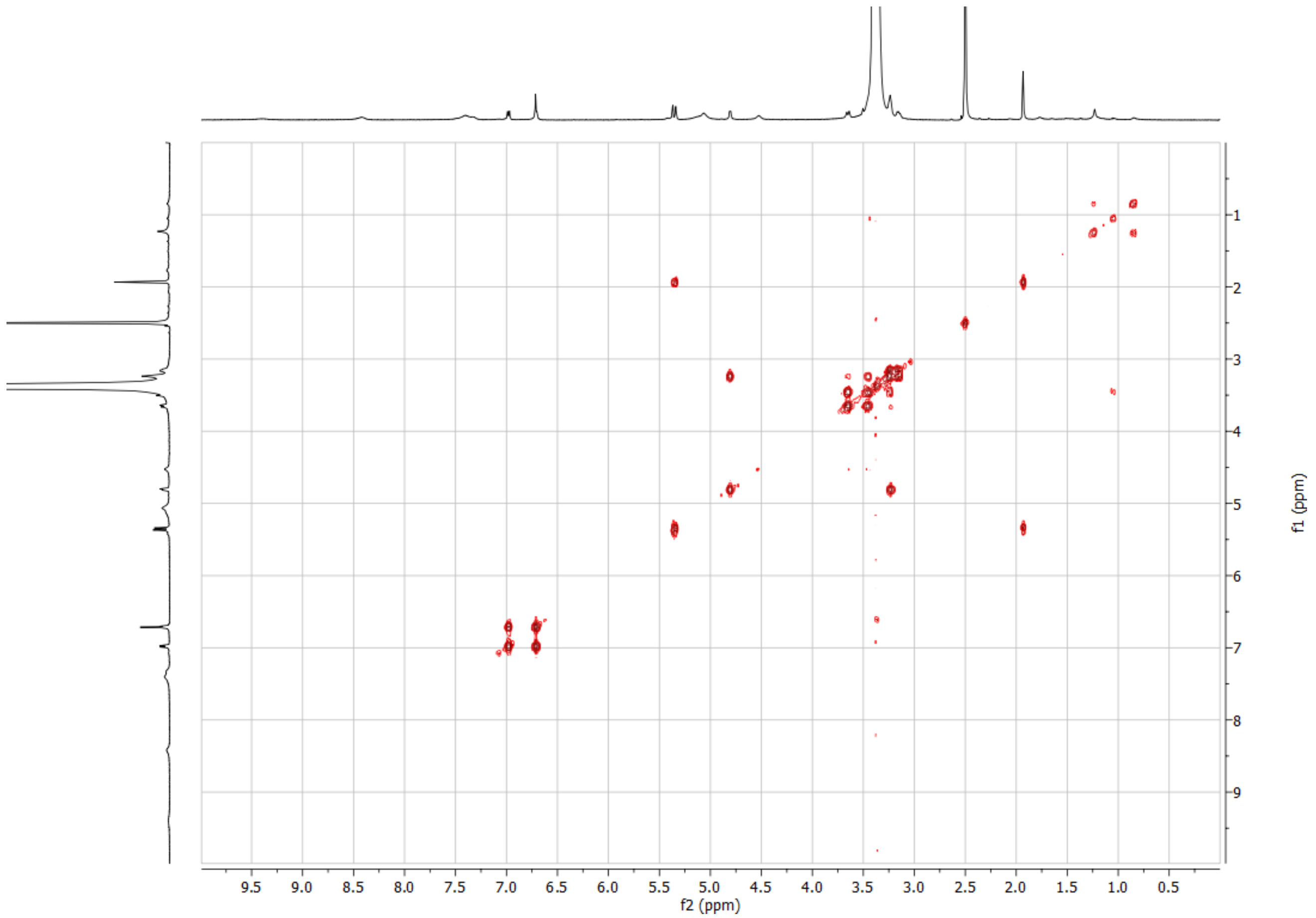

Figure S6. ^{1}H-^{1}H COSY spectrum of **1** in DMSO-d_6.

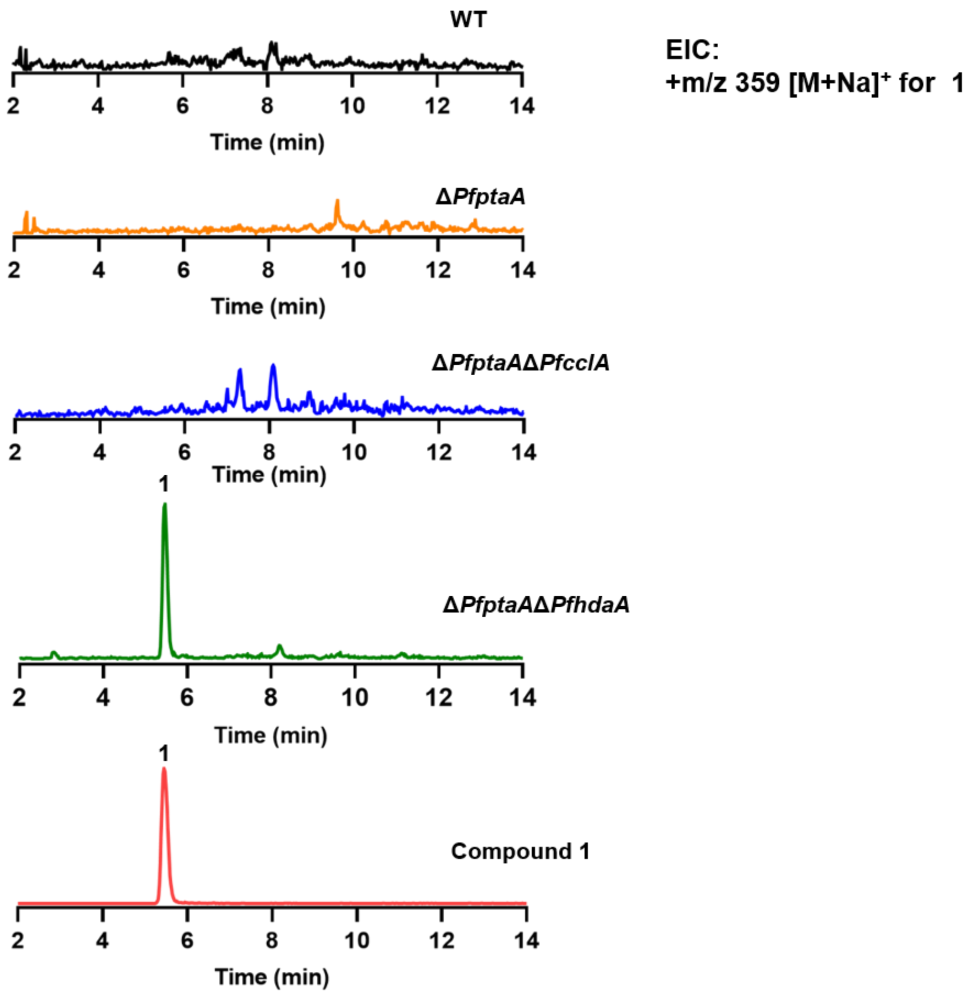

Figure S7. LC-MS analysis of compound **1** in the WT, Δ*PfptaA*, Δ*PfptaA*Δ*PfcclA*, and Δ*PfptaA*Δ*PfhdaA* strains.

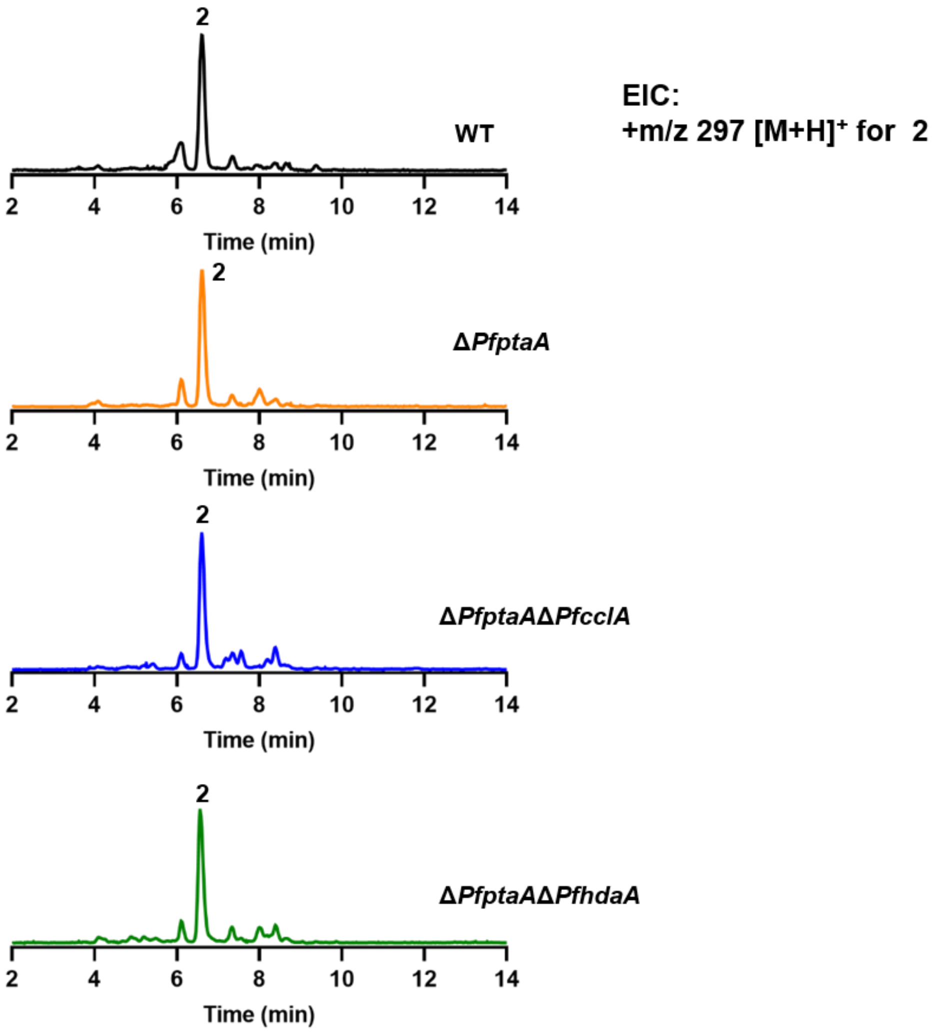

Figure S8. LC-MS analysis of compound **2** in the WT, Δ*PfptaA*, Δ*PfptaA*Δ*PfcclA*, and Δ*PfptaA*Δ*PfhdaA* strains.

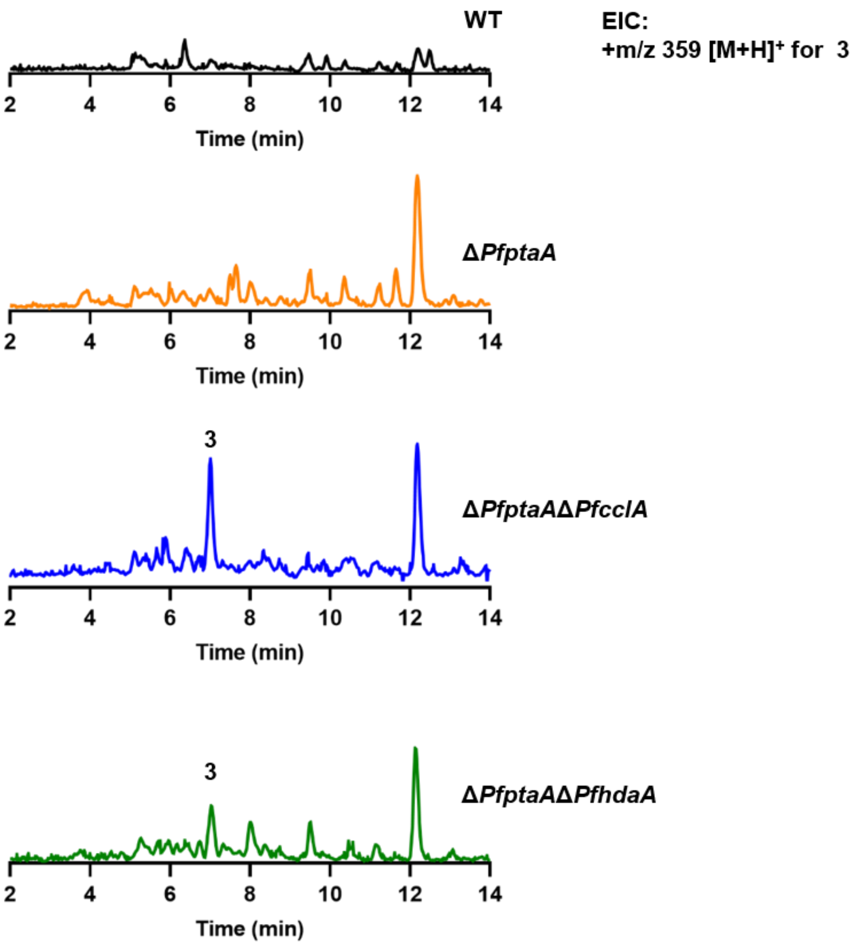

Figure S9. LC-MS analysis of compound **3** in the WT, Δ*PfptaA*, Δ*PfptaA*Δ*PfcclA*, and Δ*PfptaA*Δ*PfhdaA* strains.

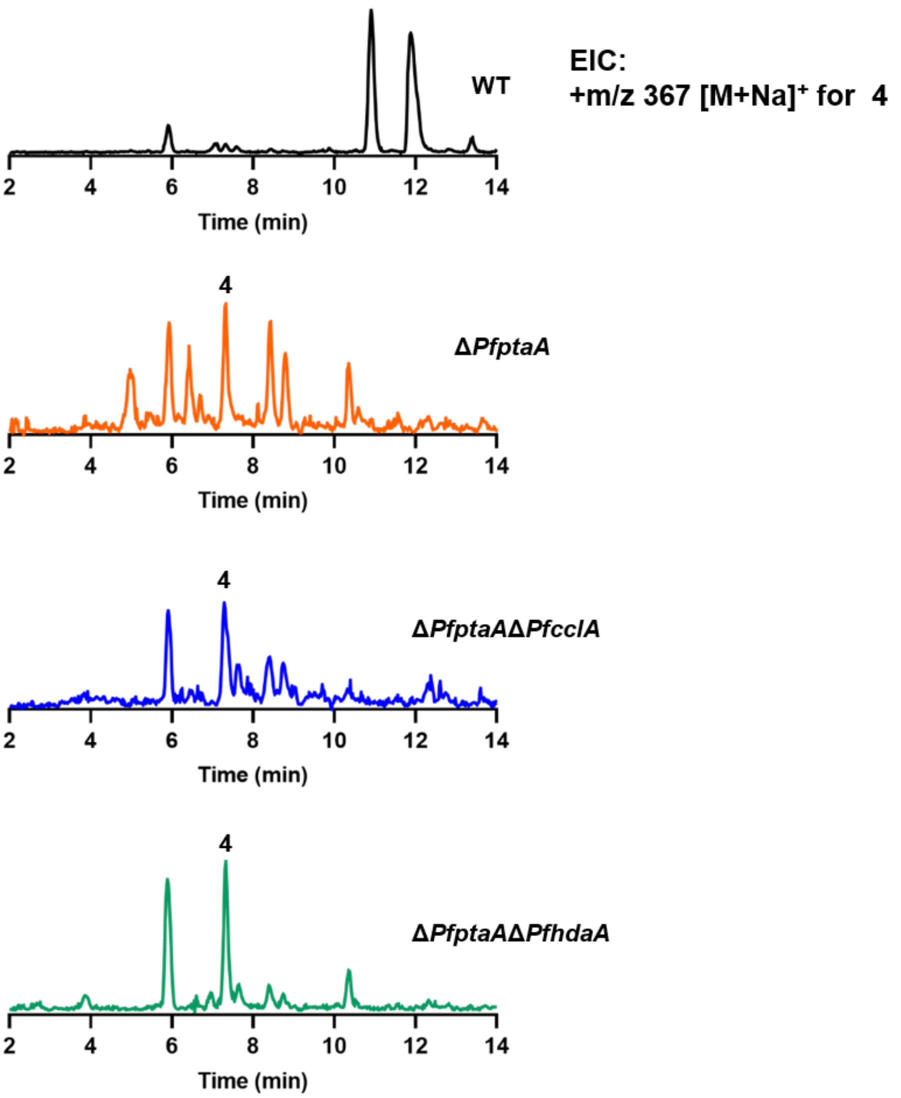

Figure S10. LC-MS analysis of compound **4** in the WT, Δ*PfptaA*, Δ*PfptaA*Δ*PfcclA*, and Δ*PfptaA*Δ*PfhdaA* strains.

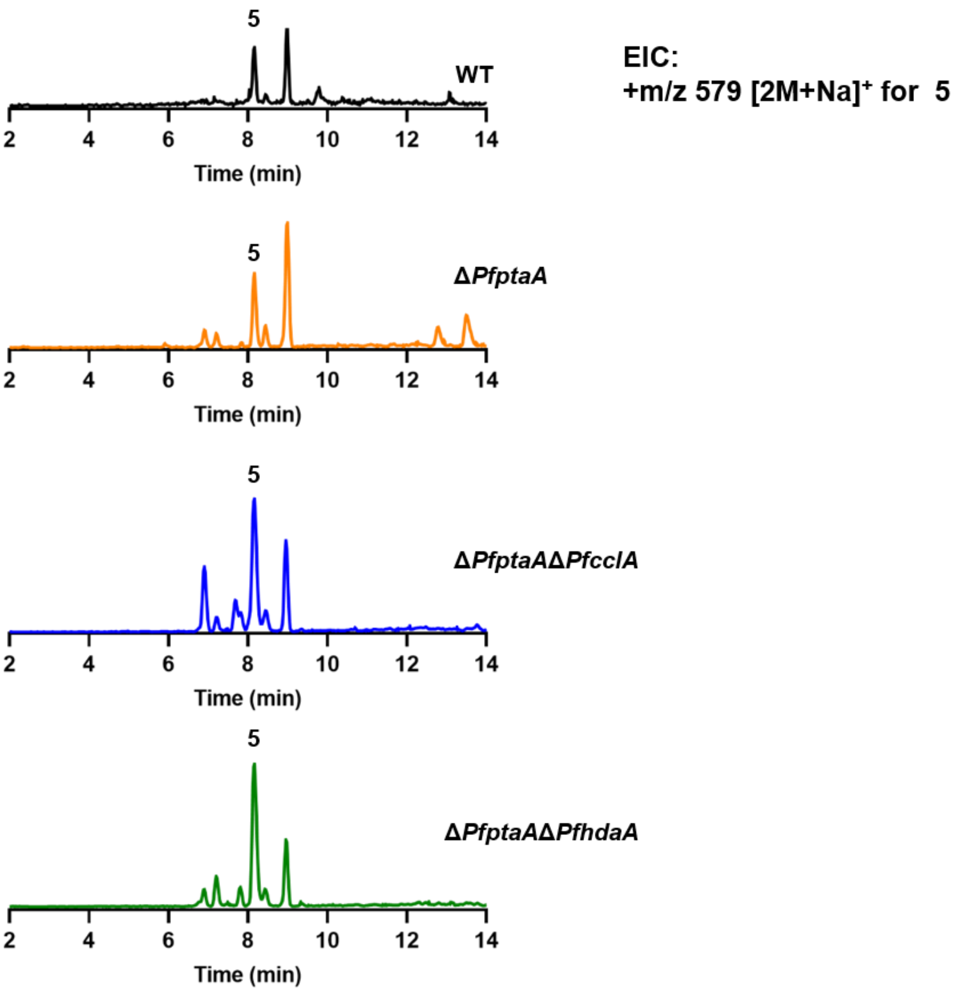

Figure S11. LC-MS analysis of compound **5** in the WT, Δ*PfptaA*, Δ*PfptaA*Δ*PfcclA*, and Δ*PfptaA*Δ*PfhdaA* strains.

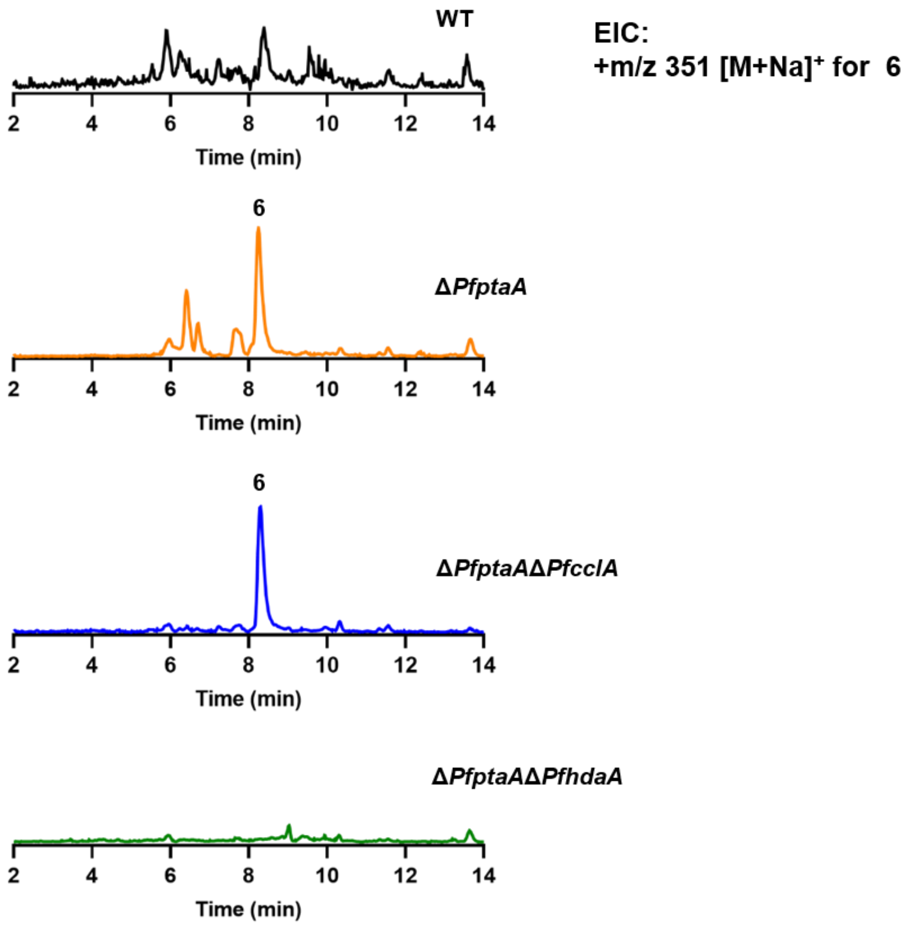

Figure S12. LC-MS analysis of compound **6** in the WT, Δ*PfptaA*, Δ*PfptaA*Δ*PfcclA*, and Δ*PfptaA*Δ*PfhdaA* strains.

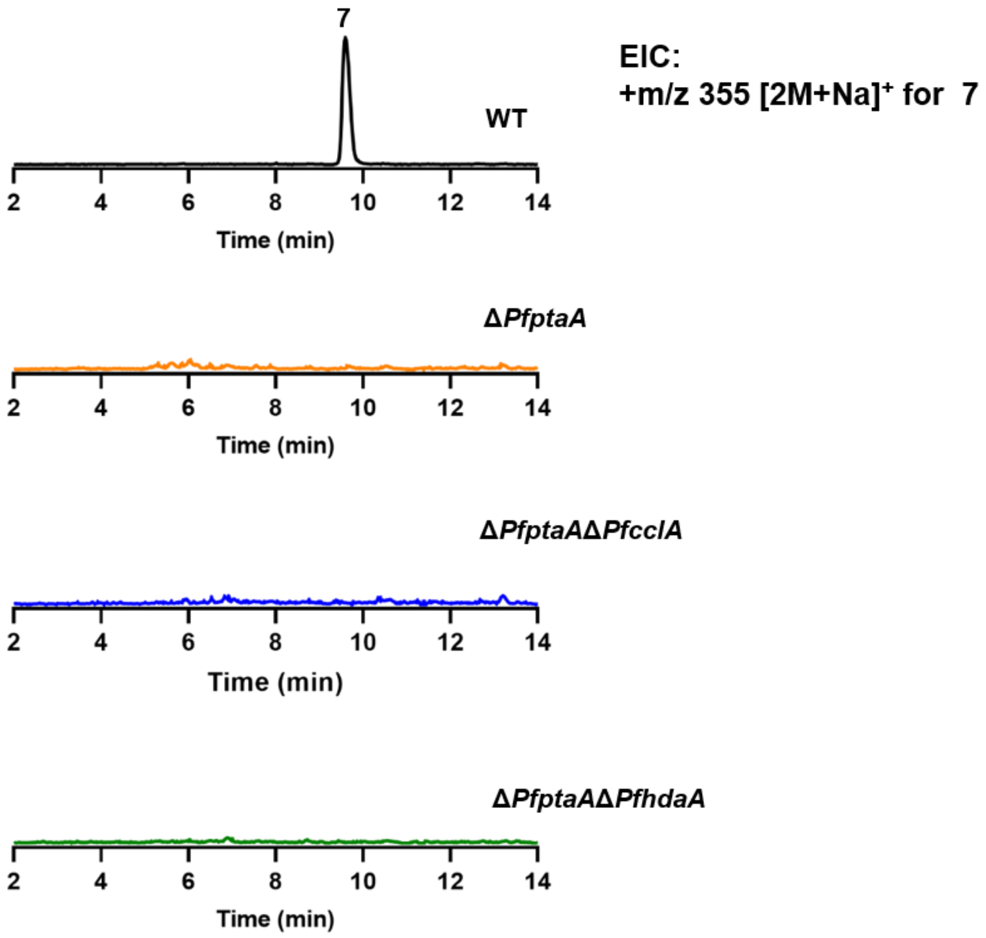

Figure S13. LC-MS analysis of compound **7** in the WT, Δ*PfptaA*, Δ*PfptaA*Δ*PfcclA*, and Δ*PfptaA*Δ*PfhdaA* strains.

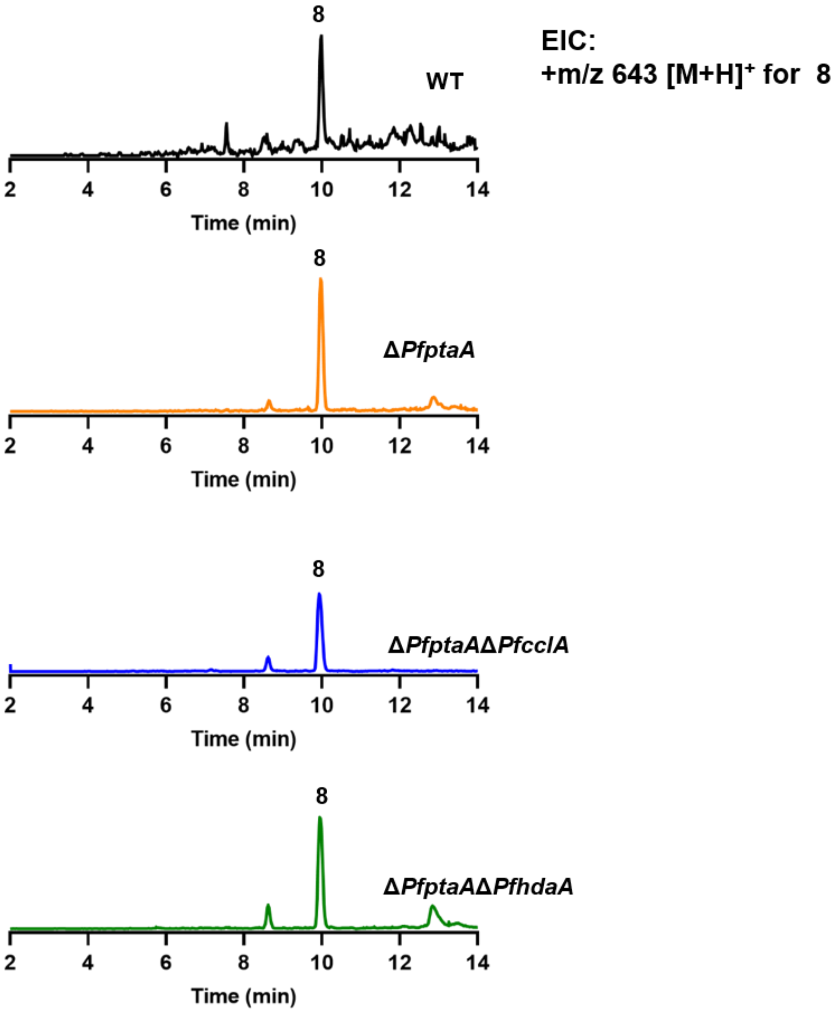

Figure S14. LC-MS analysis of compound **8** in the WT, Δ*PfptaA*, Δ*PfptaA*Δ*PfcclA*, and Δ*PfptaA*Δ*PfhdaA* strains.

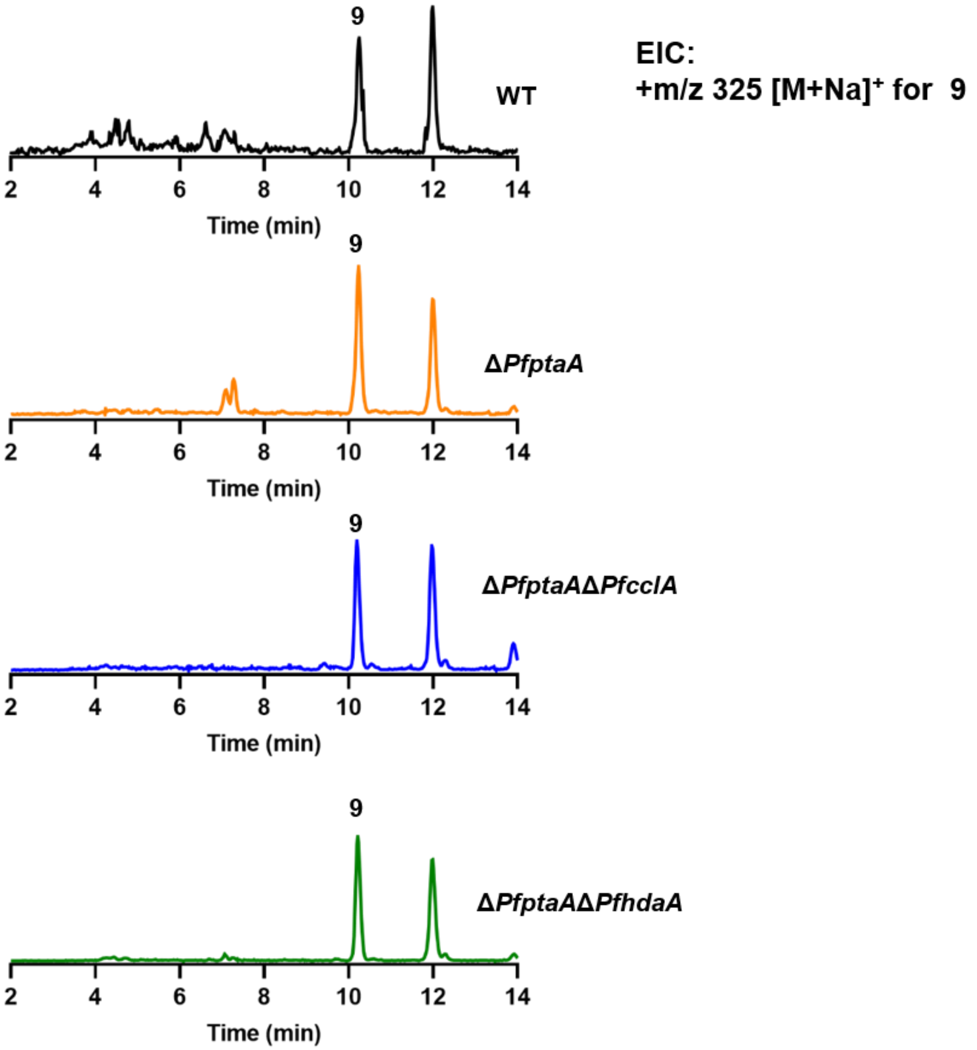

Figure S15. LC-MS analysis of compound **9** in the WT, Δ*PfptaA*, Δ*PfptaA*Δ*PfcclA*, and Δ*PfptaA*Δ*PfhdaA* strains.

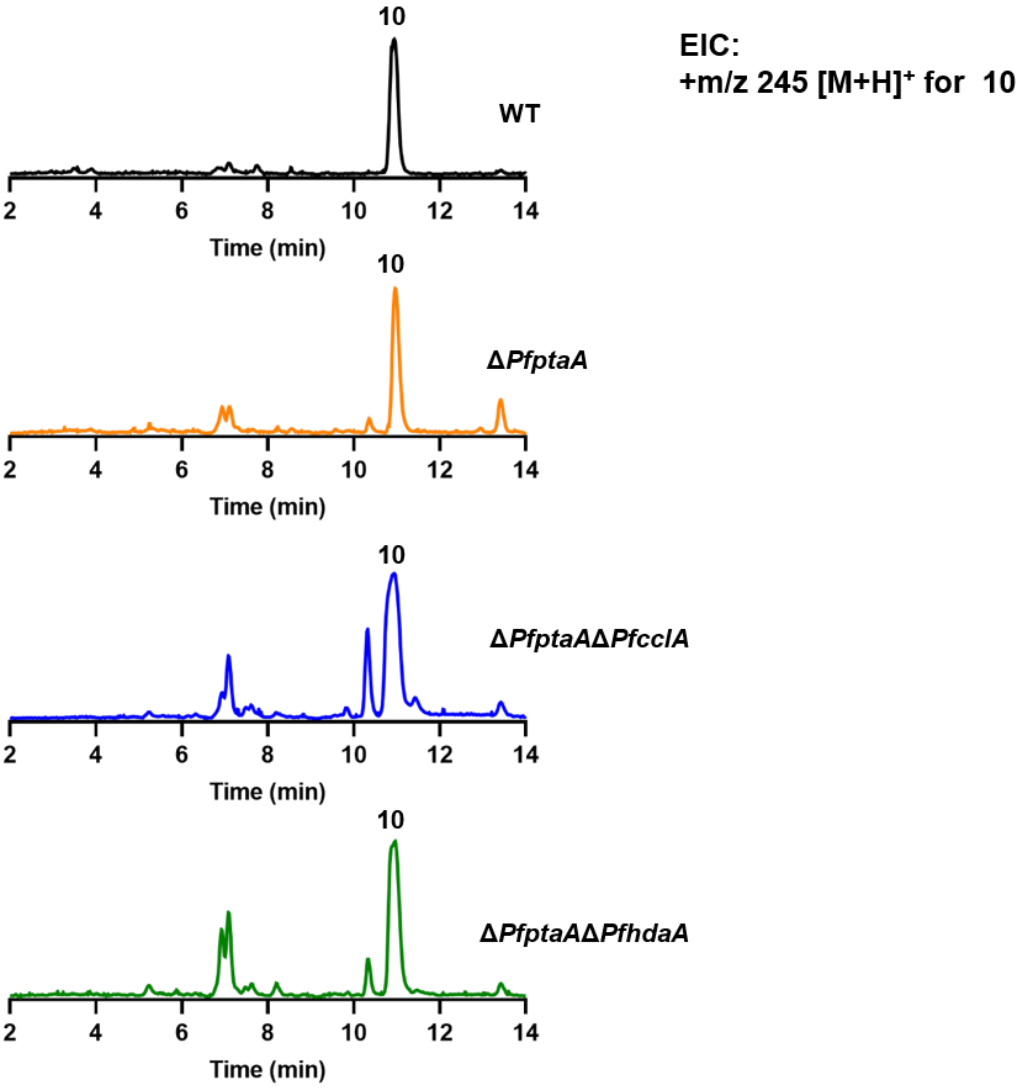

Figure S16. LC-MS analysis of compound **10** in the WT, Δ*PfptaA*, Δ*PfptaA*Δ*PfcclA*, and Δ*PfptaA*Δ*PfhdaA* strains.

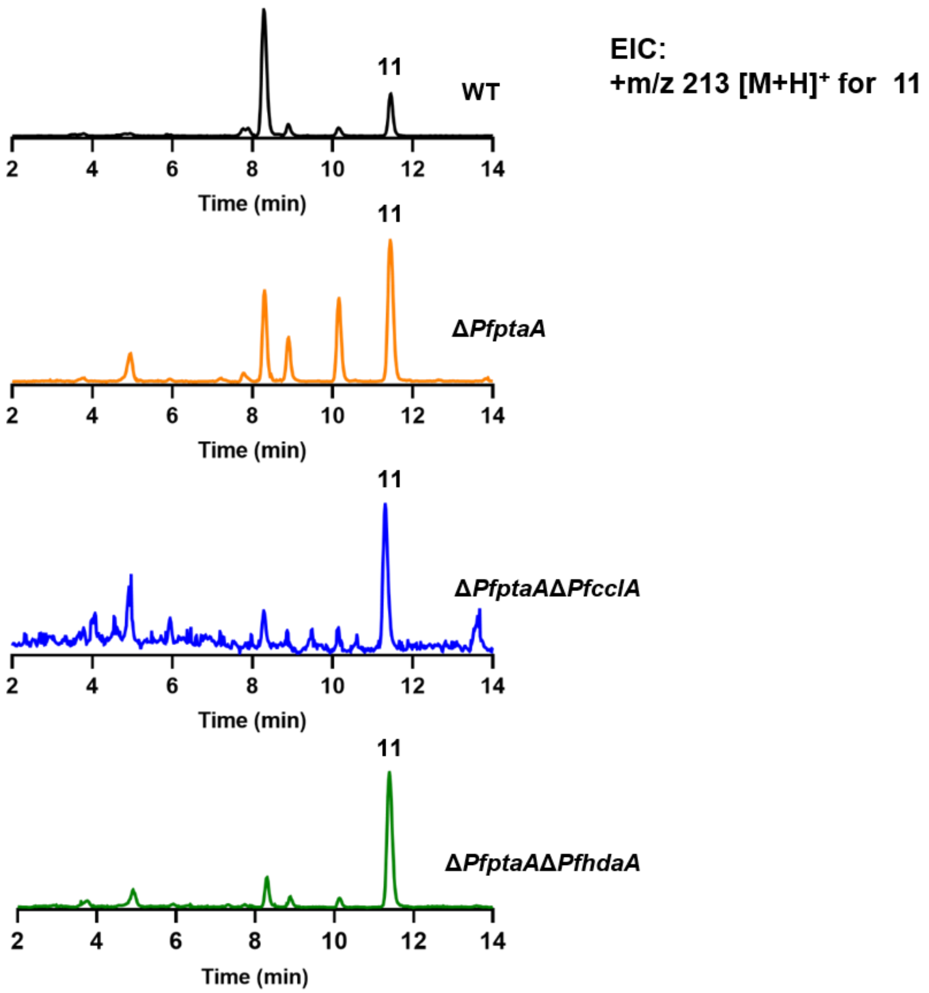

Figure S17. LC-MS analysis of compound **11** in the WT, Δ*PfptaA*, Δ*PfptaA*Δ*PfcclA*, and Δ*PfptaA*Δ*PfhdaA* strains.

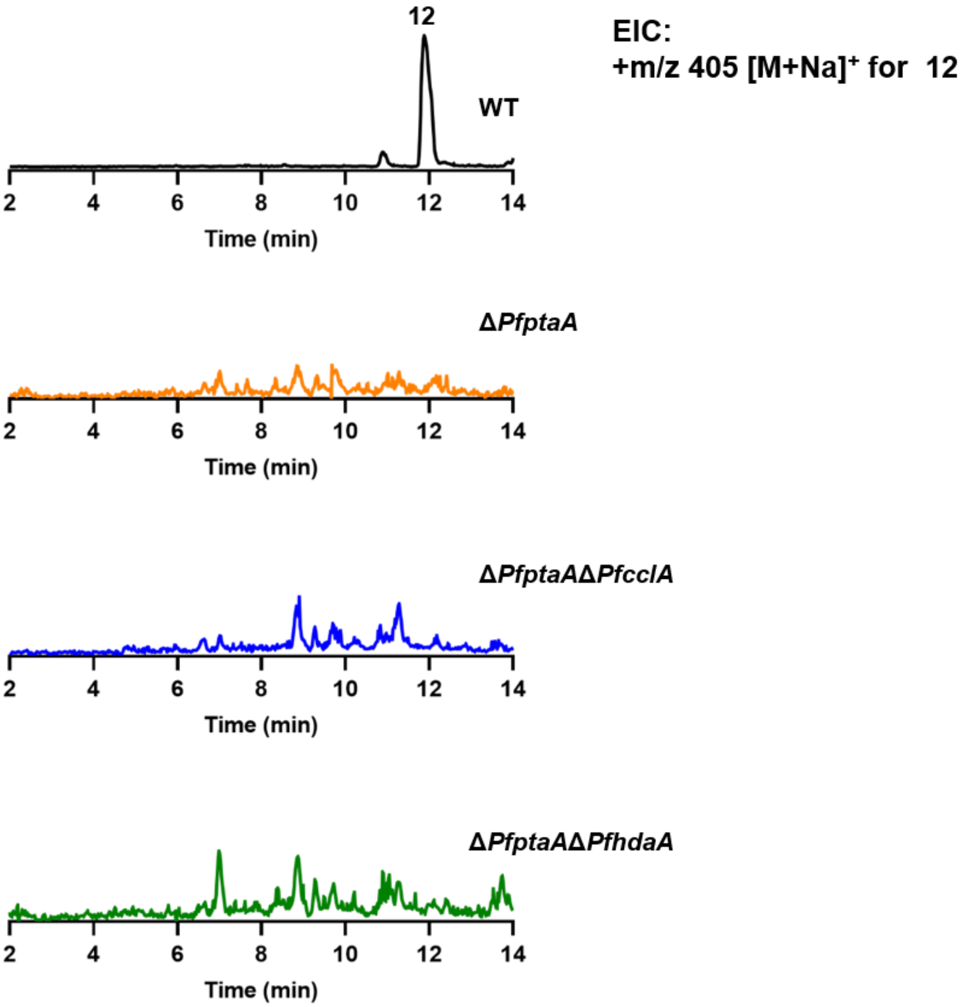

Figure S18. LC-MS analysis of compound **12** in the WT, Δ*PfptaA*, Δ*PfptaA*Δ*PfcclA*, and Δ*PfptaA*Δ*PfhdaA* strains.

References

1. Liu, L.; Liu, S.; Jiang, L.; Chen, X.; Guo, L.; Che, Y., Chloropupukeananin, the first chlorinated pupukeanane derivative, and its precursors from *Pestalotiopsis fici*. *Org Lett* **2008,** 10, (7), 1397-1400.
2. Wu, G.; Zhou, H.; Zhang, P.; Wang, X.; Li, W.; Zhang, W.; Liu, X.; Liu, H. W.; Keller, N. P.; An, Z.; Yin, W. B., Polyketide production of pestaloficiols and macrodiolide ficiolides revealed by manipulations of epigenetic regulators in an endophytic fungus. *Org Lett* **2016,** 18, (8), 1832-1835.
3. Zheng, Y.; Ma, K.; Lyu, H.; Huang, Y.; Liu, H.; Liu, L.; Che, Y.; Liu, X.; Zou, H.; Yin, W. B., Genetic manipulation of the COP9 signalosome subunit PfCsnE leads to the discovery of pestaloficins in *Pestalotiopsis fici*. *Org Lett* **2017,** 19, (17), 4700-4703.
